# Construction and evaluation of yeast expression networks by database-guided predictions

**DOI:** 10.15698/mic2016.06.505

**Published:** 2016-04-21

**Authors:** Katharina Papsdorf, Siyuan Sima, Gerhard Richter, Klaus Richter

**Affiliations:** 1 Center of integrated protein science at the Technische Universität München, Department Chemie, Lichtenbergstr. 4, 85748 Garching, Germany.; 2 Address: gerhard.richter@richterlab.de

**Keywords:** microarray, bioinformatics, yeast, S. cerevisiae, SPELL, expression networks, coregulation, clustering, GO-term, transcription factor, genechip

## Abstract

DNA-Microarrays are powerful tools to obtain expression data on the genome-wide scale. We performed microarray experiments to elucidate the transcriptional networks, which are up- or down-regulated in response to the expression of toxic polyglutamine proteins in yeast. Such experiments initially generate hit lists containing differentially expressed genes. To look into transcriptional responses, we constructed networks from these genes. We therefore developed an algorithm, which is capable of dealing with very small numbers of microarrays by clustering the hits based on co-regulatory relationships obtained from the SPELL database. Here, we evaluate this algorithm according to several criteria and further develop its statistical capabilities. Initially, we define how the number of SPELL-derived co-regulated genes and the number of input hits influences the quality of the networks. We then show the ability of our networks to accurately predict further differentially expressed genes. Including these predicted genes into the networks improves the network quality and allows quantifying the predictive strength of the networks based on a newly implemented scoring method. We find that this approach is useful for our own experimental data sets and also for many other data sets which we tested from the SPELL microarray database. Furthermore, the clusters obtained by the described algorithm greatly improve the assignment to biological processes and transcription factors for the individual clusters. Thus, the described clustering approach, which will be available through the ClusterEx web interface, and the evaluation parameters derived from it represent valuable tools for the fast and informative analysis of yeast microarray data.

## INTRODUCTION

As an efficient tool for analyzing gene expression changes, DNA microarrays are widely used to decipher reactions to environmental changes and consequences of genetic alterations in yeast, plants and humans 1,2. DNA microarrays can be used to define the expression of genes, to evaluate regulation between multiple genes, and to classify the genes according to their function and location. This is helpful in creating fingerprints of a tissue or an organ, in identifying therapeutic drug targets and in toxicology studies 3,4,5,6. Results from these analyses are lists of genes differentially expressed upon comparison of two experimental conditions (referred to as "hits"). Due to the large amount of data, computational tools are used to facilitate every step of the analysis process 7,8. Very powerful software packages are available to help with technical aspects of the method, which requires reliable analysis of the raw arrays and the derived signals 9,10. For comparison of different experiments, tools are available for cross-genechip normalization, for obtaining differentially expressed genes or for comparing different experimental conditions regarding common transcriptional responses 11. Further tools include functions to facilitate the assembly of data analysis routines such as limma 12, web servers for microarray analysis, such as ArrayMining 13 and tools to perform technical aspects of the analysis, such as TM4 14. Also methods are available to group genes according to their function, regulation or biological processes with Gene Set Enrichment Analysis (GSEA) 15,16 or clustering of the hits based on their co-regulation with Genesis 17, L2L 18 or LOLA 19. Some of these tools include databases, which are designed for specific organisms or topics, in many cases for human and mammalian samples 15,18,19.

Clustering differentially expressed genes according to their transcriptional connection in most cases is performed by simultaneous analysis of larger numbers of microarray experiments 13,17,20. This approach is very powerful to get information on common patterns observed in many arrays. It requires substantial input of array data and, thus, requires that sufficient numbers of experiments have been performed or arrays from the public domain are included in the experiment as training sets. Many studies have convincingly shown the power of such approaches and the advantages to apply these methods for the construction of co-expression networks. For studies with very few arrays, this can make the selection of the training set and the data handling difficult, as the clustering results may be influenced by the selection of these data sets.

We previously had set out to describe the transcriptional response to polyglutamine-induced toxicity 21. Performing analyses on a sample set of only six arrays to compare three conditions for an unknown response, the selection of training sets was challenging. We thus used the extensive co-regulation information from the SPELL (Serial Pattern of Expression Levels Locator) database, which is based on the analysis of more than 10,000 microarray experiments. By correlating the results from our DNA microarray experiment with this database, we visualized the networks of the transcriptional responses to polyglutamine-induced toxicity and obtained informative networks for each condition compared 21. Each of these networks was constructed based on the 100 strongest differentially expressed genes. Here, we statistically evaluate and further develop this database-guided clustering method for yeast microarray data. We define the sensitivity of the calculated networks to changes in the input parameters and define options to score the validity of the networks based on connections and predictions, which can be obtained from the connected hits.

## RESULTS

### Microarray data sets can be clustered into interconnected networks based on publicly available co-regulation data.

**Figure 1:**
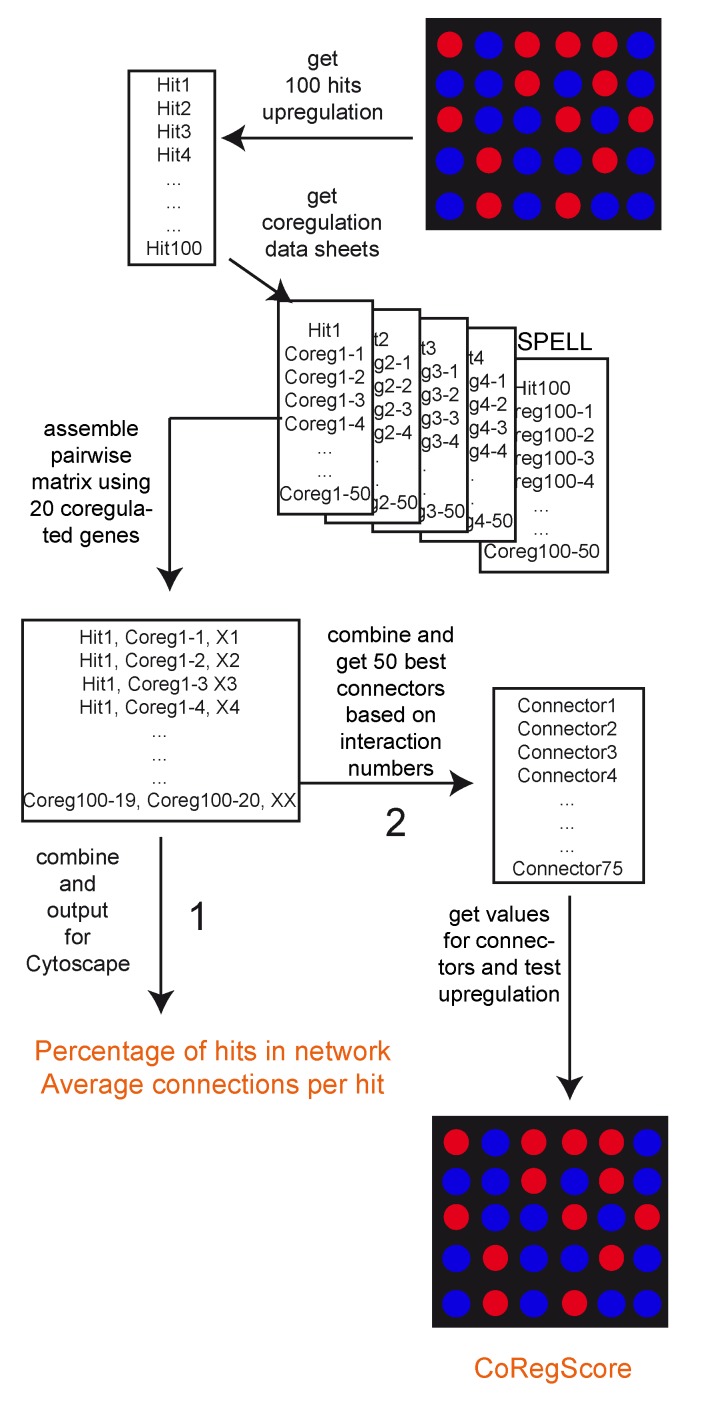
FIGURE 1: Data processing in ClusterEx. A flexible number of hits (here the top 100) are obtained from the microarray data set as "hits". For each hit, the best co-regulators (here 50) are obtained from the SPELL database. These are assembled into a pairwise matrix. After combining, each pair is only listed once with the number of occurrences (X). This matrix then is exported to Cytoscape and values are reported for percentage of hits included in the network and connection numbers per hit (Pathway 1). Furthermore, a flexible number (here 50) of connectors from the matrix can be included into the network. For those the real expression values are obtained from the experimental data set. The CoRegScore is calculated from the positioning of these predicted connectors in the hit list of the experiment (Pathway 2). This procedure is described in the materials and methods section.

**Figure 2:**
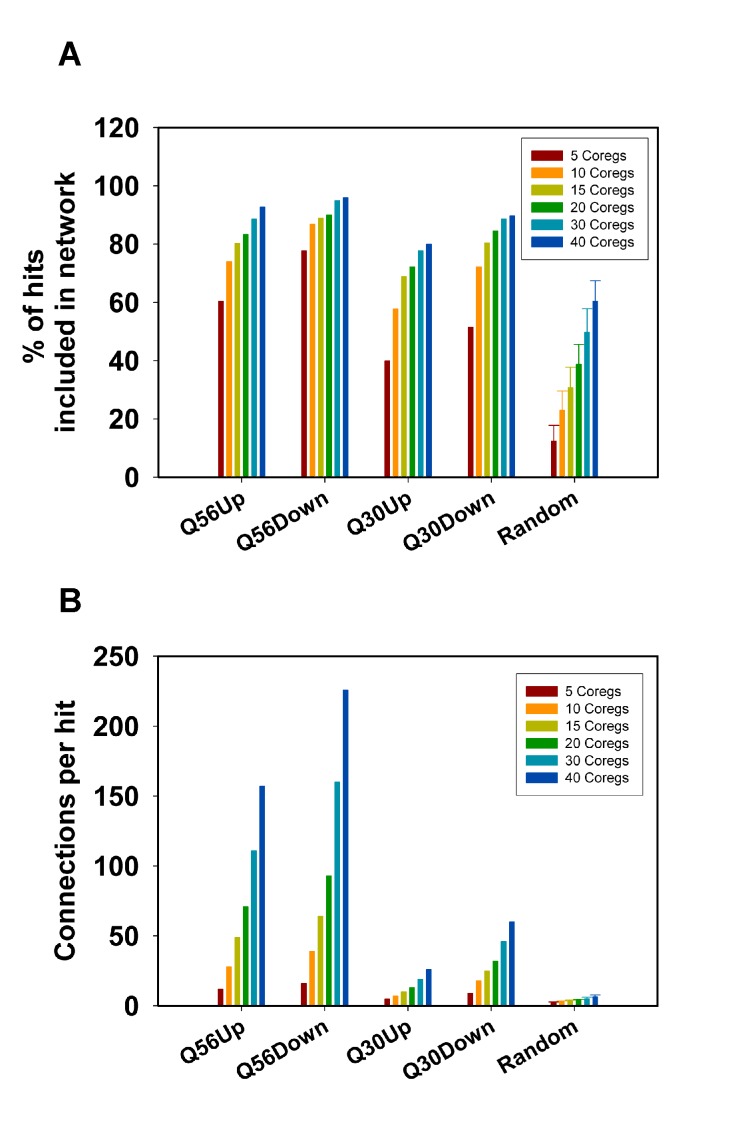
FIGURE 2: Analysis of network connectivity based on the used number of co-regulated genes. Connectivity parameters for hits derived from the Q56- and Q30 datasets were calculated for 100 hits. The best 5-40 co-regulators were obtained from the SPELL data sheets. **(A)** Depicted are the number of hits, which are included in the network and **(B)** the average number of connections per hit. As a control 100 random sets of genes were used and processed in the same manner. The error bars represent the standard deviation for the 100 random gene lists.

We recently reported on the transcriptional response of yeast cells to the overexpression of toxic polyglutamine proteins 21. Here, toxic polyglutamine stretches contain-ing 56 residues (Q56) and non-toxic stretches with 30 residues (Q30) were expressed in yeast and compared to a non-polyglutamine expressing control. This approach results in several hundred genes, which are up- or down-regulated in a single microarray experiment. We clustered these genes to visualize potential transcriptional networks (Figure 1) by using information about co-regulatory relationships from the SPELL webserver 22. This data resource contains information on co-regulated genes, providing 50 co-regulated genes (called co-regulators throughout the manuscript) ranked according to their correlation score in each single-gene query. To assess the sensitivity of the networks generated by this approach, we first tested the quality of the resulting networks, if we cluster the genes of the hit list using the information from varying numbers of co-regulators. As such, we initially used the 100 most differentially regulated genes (called top100 throughout the manuscript) and clustered them based on 5, 10, 15, 20, 30 or 40 co-regulators from SPELL. To evaluate the networks we determined the number of hits, which incorporate into a network by having at least one network connection to another hit (Figure 2A). Indeed, we observe that most of the top100 hits can be connected into a network, suggesting that the top100-hits from our microarray experiment are part of a transcriptional response, which correlates with the information in SPELL. This holds true for both directions in our experiments.

To get statistical confirmation on these dependencies we used 100 sets of equally sized random hit lists and determined to what extent our experimental microarray data perform better compared to the random hit lists. The random hit lists show significantly less connected genes in the network with p-values under all conditions below 1E-05. Visualizing the obtained networks in Cytoscape 23 it also is obvious that most connected genes in the random networks only have one or two interaction partners (Figure S1). We additionally determined the number of connections per gene to further prove that the connection numbers are significantly better for our experimental set compared to random gene lists. This is the case for every number of SPELL-derived co-regulators and the differences are significant with p-values below 1E-05 for all of them. In fact, if 40 co-regulators are used to construct the network matrix, random hit lists yield approximately 12 connections per hit compared to more than 200 for the top100-hits down-regulated in response to Q56-expression (Figure 2B). Visualizing the networks, the differences between the two polyglutamine experiments become obvious, with one of them (Q30 vs. Q0) being more loosely connected, while the response in the other network (Q56 vs. Q0) strongly clusters (Figure S1).

It is also evident from this approach that the number of included hits and the number of connections per hit increase with the number of co-regulators used to build the matrix. This is true for all Q56 and Q30 datasets (Figure 2A, 2B) – but this is also true for the random gene lists (Figure 2A, 2B), implying that this approach could principally lead to clustering into large networks even of random genes, if too many co-regulators are used to build the network matrix.

**Figure 3:**
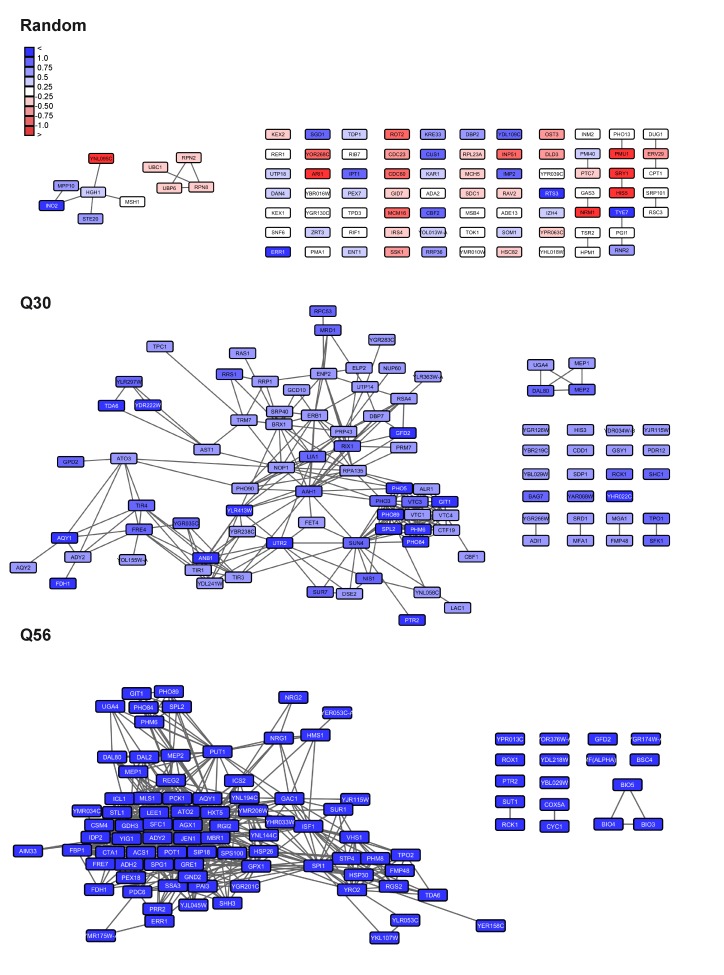
FIGURE 3: Analysis of network connectivity based on the number of initial hits. Connections between hits derived from the Q56- and Q30 datasets were calculated based on a variable number of hits. The top30-top300 most strongly regulated hits were used to construct a network. **(A)** Depicted are the number of hits, which are included in the interconnected network and **(B)** the average number of connections per hit. As a control 100 random sets of genes were used and processed in the same manner. The error bar represents the standard deviation for the 100 random gene lists.

We also tested to what extend the size of the initial hit list influences the connectivity parameters for the polyglutamine datasets. We calculated the networks for the polylgutamine data sets and for random hit lists using 20 co-regulators and this time varied the number of hits. The percentage of included hits and the number of connections per hit was calculated, if instead of the top100-genes 30, 50, 75, 125, 150, 200 or 300 hits are used for network construction (Figure 3). The percentage of hits connected into these networks increases for the random hit lists, if the size of the lists gets larger (Figure 3A). At 300 genes, these lists already contain about 5 % of the yeast genome, making it very likely that a co-regulated partner for an individual hit is contained in the hit lists. Regarding the number of connections per hit also the four experimental datasets show significantly higher readings compared to the random hit lists (p-values for 300 hits: < 1E-05 for all). In the random data most hits again have only one or two interaction partners with one or two connections (Figure 3B). For the Q56 and Q30 data sets, this type of database-guided clustering clearly exposes the most important transcriptional networks composed of differentially expressed genes. But for both described evaluation parameters – the number of hits in the network and the number of connections per hit – the usage of large numbers of co-regulators or hits eventually will lead to results, where the size of the random networks gets more similar to the networks based on experimental results. This implies that selecting a reasonable number of hits and co-regulators is important to obtain significant results. We chose to include the top100-hits and 20 co-regulators as well-balanced starting point.

### Addition of co-regulators predicted from databases increases the connectivity of the network.

When we inspected the matrix containing all network connections we realized that many genes - even though they are not part of our top100-list – are highly connected to our top100-hits and thus are co-regulated in the SPELL-database. Also in many cases these genes are indeed differentially expressed, but just not strong enough to be part of the top100 genes we included in the hit list for network analysis. This points to a remarkable ability of this network construction method to correctly predict further co-regulated genes based on the connections within the network matrix.

As a third parameter for network significance we aimed at deriving a score which quantifies this predictive ability of the network. To this end new genes, which are highly connected in the network, were obtained from the connection matrix and exported as “list of predicted new co-regulated genes” (called connectors herein). To visualize their connection to the top100-hit genes we included the connectors into the network (marked by a grey frame). This has two positive consequences: first, it could include so far isolated hits into the network in a meaningful way. Second, the predictive strength of the network in general could be assessed based on these connectors by comparing the prediction with the real expression differences.

We thus calculated networks of top100-hits from our microarray experiments as described before using 20 co-regulated genes for each of them and expanded the networks by a maximum of 10, 20, 30, 40, 50, 75 or 100 connectors derived from the network matrix based on their high number of connections to the top100-hits. After including these connectors we again calculated the percentage of included hits. Indeed, we observe that some previously isolated genes from the hit list became part of the network when connectors are included (Figure S2A). Likewise the number of connections per hit increases with higher numbers of connectors in the network (Figure S2B). The inclusion of these connectors thus indeed improves the quality and information content of the networks.

### A scoring function can assess the accuracy of predicted co-regulators and provide statistical information about network quality.

**Figure 4:**
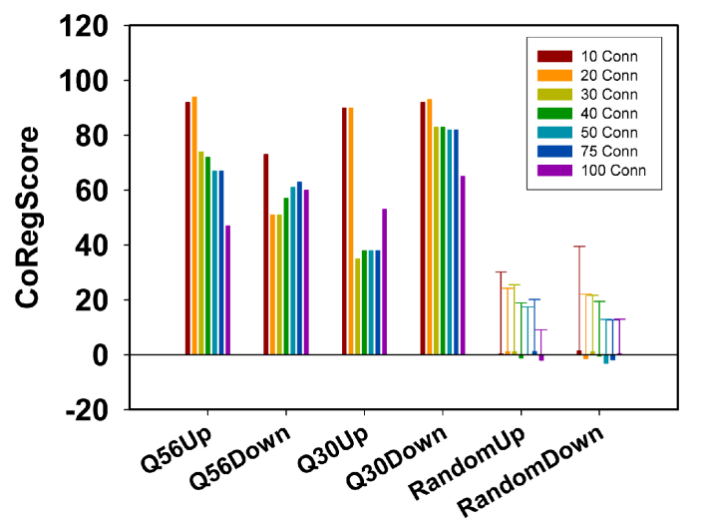
FIGURE 4: Analysis of connectors based on their real expression behavior. The CoRegScore evaluates to what extent connectors are regulated in the predicted direction. A positive score implies that the majority of algorithm-determined connectors are regulated in the same direction as the top100-hits. To obtain a CoRegScore of 100, the predicted connectors have to be the next genes after the top100 in terms of expression differences. The CoRegScore is calculated as described in the materials and methods section. Depicted here is the variability of the CoRegScore depending on the number of connectors derived from the pairwise co-regulation matrix. 100 random experimental sets were constructed and evaluated under the same conditions to obtain the average score of a random data set and the standard deviation of this value.

So far evaluation of the networks was based on the percentage of included hits and the number of connections per hit. After determination of these supposedly co-regulated connectors, it now is possible to use the quality of these predictions to quantify the predictive ability of the network. This can be achieved by comparing the predictions with the real expression values for these connectors. If they are indeed part of the transcriptional clusters as predicted by our clustering routine, they should be regulated in the same direction in this microarray experiment. We thus define and implement a scoring function in our ClusterEx routine, which describes the quality of these predictions. This can be done by sorting all 5,815 genes from the array data set according to their expression differences. The first 100 genes are identical to the top100-list used to create the network. If the 50 predicted connectors would be 101-150 in the sorted list of all array genes, the prediction would be perfect. We thus use the positioning of the connectors in the sorted list to determine a score (called CoRegScore) from 100 (best possible positioning of the connectors) to -100 (worst possible posi-tioning of the connectors) and obtained these scores for our polylgutamine microarray data sets in the analysis. Indeed, all scores are clearly positive with values of 68 (Q56up), 59 (Q56down), 38 (Q30up) and 80 (Q30down), if 50 connectors are included (Figure 4).

To assess, whether these values show a statistically significant deviation from random data, we performed the calculation of the CoRegScore on randomly generated array data. Indeed, an average CoRegScore close to 0 was obtained after evaluating the 100 random array sets. The standard deviation of these random gene sets drops with the number of connectors included, suggesting that best results can be obtained, if about 50 connectors are derived for a network constructed as described (top100-hits with 20 SPELL-derived co-regulators). p-values under this condition are determined to be 5.9E-05 (Q56up), 2.3E-05 (Q56down), 0.0144 (Q30up), and <1E-05 (Q30down), implying that the predictive ability of the Q30up direction is the weakest. This correlates also with the lower number of connections under this experimental condition and hints at a network, which may not significantly represent a coordinated transcriptional response. For the other three networks instead, most predicted connector genes are indeed regulated in the same direction as the rest of the network they were calculated from. This shows that the co-regulated clusters exposed by the algorithm behave predictable and thus likely represent transcriptional units in which all member genes respond as part of the response.

### Statistical evaluation of expression networks is generally applicable for yeast microarray datasets.

**Figure 5:**
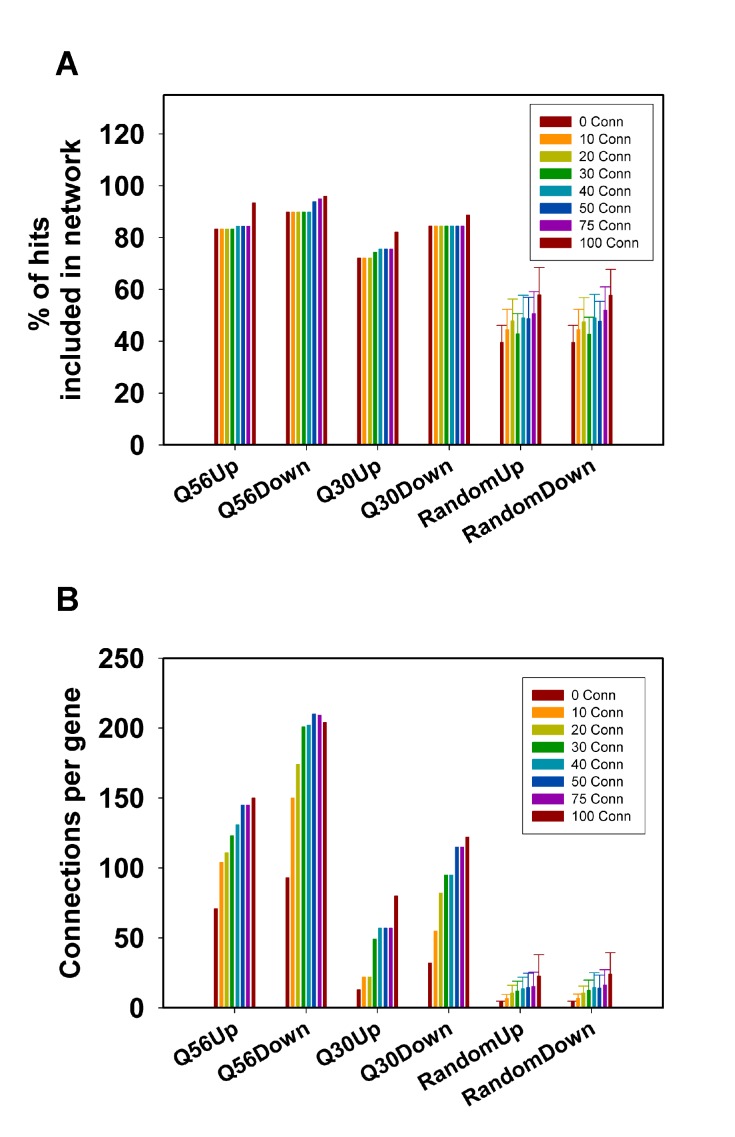
FIGURE 5: Evaluation of parameters for different experimental setups. Different microarray experiments were used to test the general applicability of the clustering approach, the connectivity parameters and the CoRegScore. **(A)** Depicted here is the percentage of hits included within the network upon analysis of the top100 connected by 20 co-regulators obtained from SPELL. **(B)** The average number of connections per hit is shown under the same conditions. **(C)** The CoRegScore is calculated for each data set after addition of up to 50 connectors and compared to the values for random data sets derived before.

Using several evaluation options we have demonstrated that the clustering of hits using SPELL-derived co-regulators in our ClusterEx algorithm generates significant networks for our polyglutamine data sets. To show that this approach is generally applicable for yeast microarray data sets, we picked ten microarray datasets from the collection of more than 10,000 experimental microarray data on the SPELL webserver. Picking ten data sets from this resource should provide unbiased information on the general functionality of the algorithm. Data sets were used from a study on the heat shock response in yeast 24, a study on the overexpression of transcription factors 25, a study on the change in nutritional conditions 26, phosphate deprivation 27 and growth inhibition by rapamycin, iodide or thyamin among others 28,29,30,31,32 (datasets summarized in Table S1). We used the same parameters as outlined before (20 co-regulators from the SPELL list for the top100-hits, maximum of 50 included connectors). We calculated for each of these the corresponding networks and determined the percentage of hits included. In all cases the percentage of included hits is at least two-fold higher compared to random gene lists (Figure 5A). We also determined the number of connections per hit and find that each of the twenty networks performed better than the random data sets (Figure 5B). Some of the networks yield more than 200 connections per hit, whereas others are not as closely connected. Nevertheless, for all those experimental data sets the described clustering approach results in a significant network structure.

**Figure 6:**
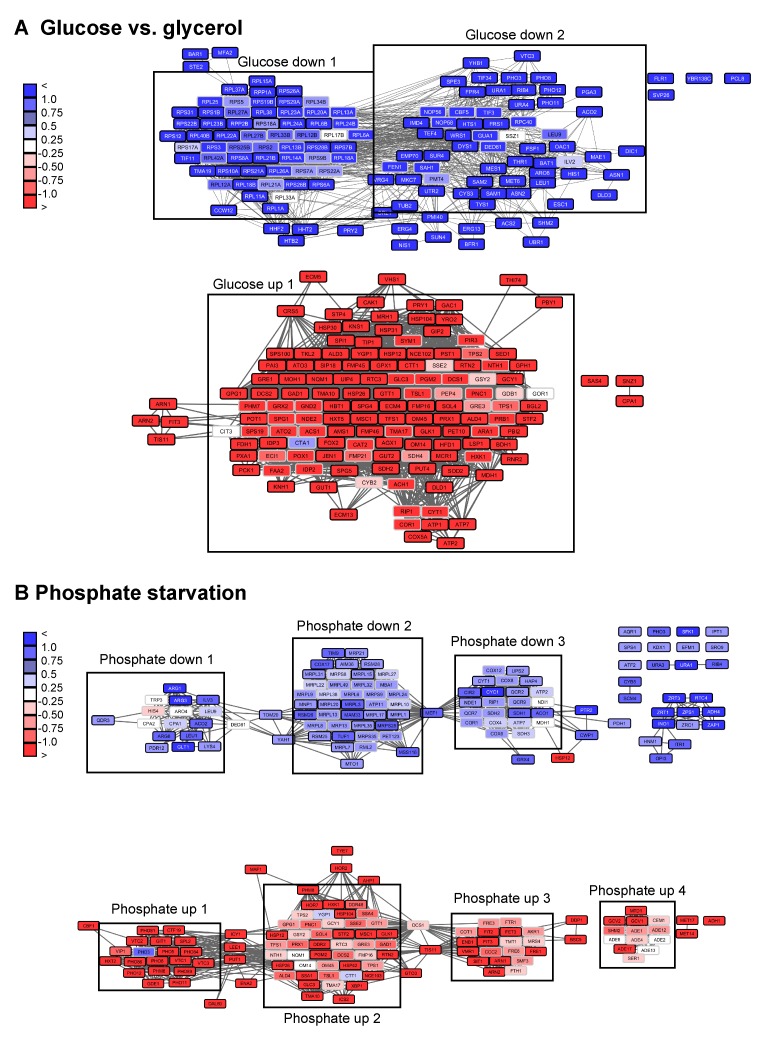
FIGURE 6: Visualization of networks upon glucose-depletion and phosphate starvation. The top 100-hits of different datasets were connected in ClusterEx and visualized in Cytoscape. Connectivity is retrieved as described by obtaining 20 coregulators from SPELL and including 50 connectors. The edge-weighted spring embedded layout is used to position highly connected genes in close proximity in Cytoscape. Genes are colored according to their log differences in the respective experiments. Clusters which are further analyzed for their GO-terms via PANTHER and for their transcription factors via the YEASTRACT web service are marked with black boxes. A) Genes differentially regulated in *S. cerevisiae* upon growth on the carbon sources glucose versus glycerol 37 were built into an interconnected network. The top100 hits (black frame) and 50 connectors (grey frame) were included into the network. Upper panel: downregulation, lower panel: upregulation. B) Genes differentially regulated in *S. cerevisiae* upon phosphate starvation 27 were built into an interconnected network. The top100 hits (black frame) and 50 connectors (grey frame) were included in the network. Upper panel: downregulation, lower panel: upregulation.

We then compute up to 50 connectors for each network and include them to quantify the predictive strength as described by the CoRegScore. All networks constructed showed values higher than 0. They yielded scores between 96 (Z-Score=5.43, p <1E-05) and 22 (Z-Score=1.25, p=0.1056). This implies that in most of these cases the co-regulation analysis as outlined in our procedure (Figure 1) yields significant correlations and extracts transcriptional clusters based on the top100 differentially regulated genes, while also providing the connections for the visual description of these networks (Figure 6). In some cases, like for the response to heat (Figure S3B) this is highly significant, while in others, like for the overexpression of Hsf1 (Figure S3A) it becomes obvious that the predictive strength is much less pronounced due to a weaker and much less orchestrated response. We thus believe that the CoRegScore and the method to derive it from database-guided clustering could be a valuable tool in defining the significance of the connections and the information content in a clustered expression network.

### Clustered expression networks provide better significance of derived biological information.

**Figure 7:**
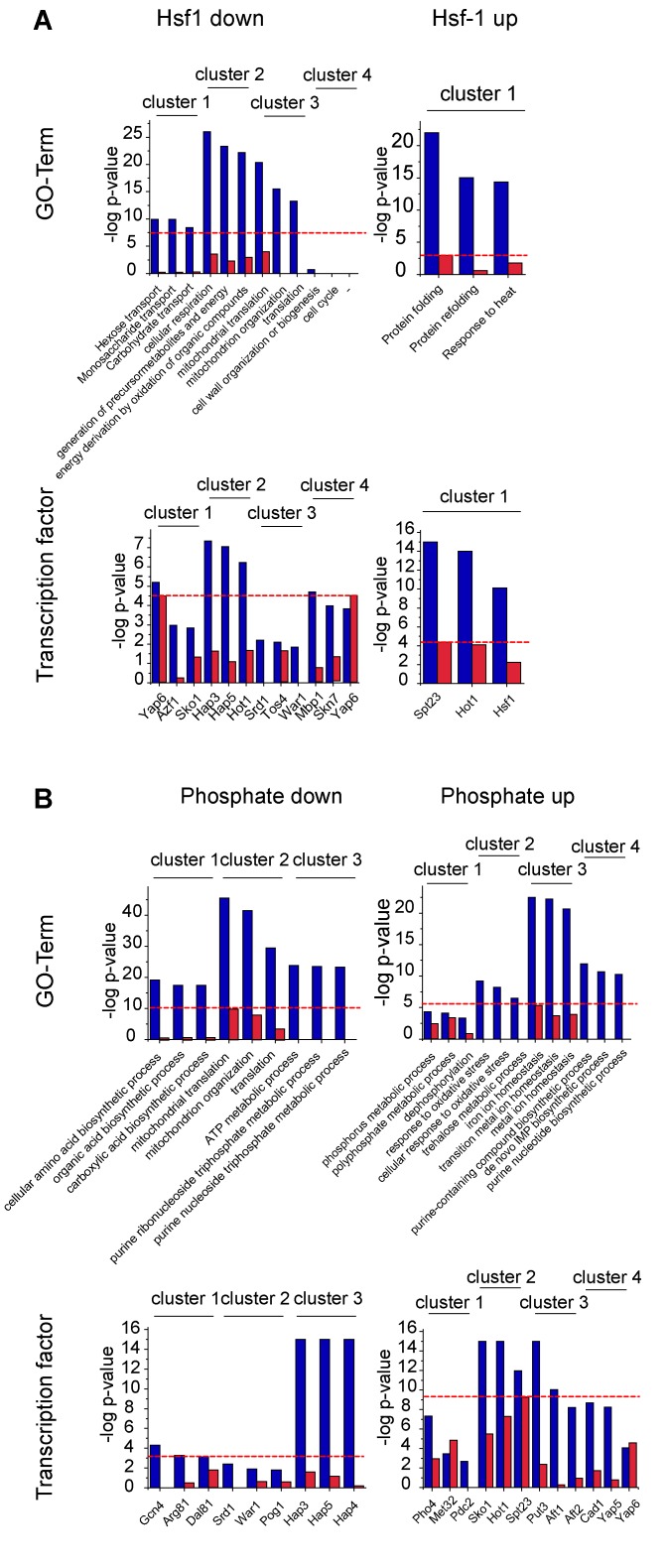
FIGURE 7: Biological processes and transcription factors of isolated clusters. Clusters are marked in Cytoscape as indicated in the corresponding figures (Figure 6, S3). The gene-ontology term (GO-term) biological process of the genes (upper panel) and the transcription factors regulating the genes (lower panel) of the isolated clusters (blue) and the full gene hit list (red) are depicted. The full analysis is listed in Table S2. The negative log of the p-value of the three highest ranking terms of the cluster analysis and the corresponding values of the full hit lists analysis are depicted. The red line marks the p-value of the best GO-term or transcription factor, when analyzing the non-clustered control of all hits. "-" = analysis did not yield in any significant results.

When visualizing the networks with Cytoscape it becomes apparent that in some cases distinct clustering units are observable in the same network. Thus, if more than one expressional cluster is part of the overall response, the described algorithm is able to assign the hits and divide them into individual parts of the response (see Figure 7). We aimed at testing, whether within these clusters significant biological information is enriched. To this end wemanually isolated clustered regions in Cytoscape and tested whether this selected group of hits provides more and better information about the functionality and the origin of the transcriptional response compared to the full unclustered hit list. To evaluate the biological information hidden within the transcriptional clusters we analyzed them according to their involved biological processes via the gene-ontology terms (GO-terms) 33,34 with the PANTHER web service 35. We further determined enrichment of binding sites for transcription factors for the hits within the clusters using YEASTRACT 36 (Table S1). Indeed, in almost all cases a striking improvement of the p values can be obtained for the isolated clusters compared to the full unclustered hit list. This shows that the full response may be composed of several reactions, which can be much better assigned to GO-terms or transcription factor dependencies based on isolated clusters (Figure 7). Notably, even for small clusters, now individual transcription factors can be assigned by YEASTRACT, which before was impossible when the full unclustered hit list was used.

The described clustering approach thereby disentangles the multifaceted cellular response. This in particular is evident for the response to phosphate starvation (Figure 6). Here, for example, we receive significant enrichment of the transcription factors Gcn4 (cluster down1, p-value: 5.0E-05) and Pho4 (cluster up1, p-value: 4.57E-08) for phosphate starvation in individual clusters, but hardly any significance, when the full list is used as query with p-values of 0.99 and 0.108 respectively (Table S2, Figure 7). The same holds true for biological processes, which are also positively affected by investigating clustered subsets compared to the full hit list (Table S2). The detailed analysis of the yeasts’ response to phosphate starvation thereby reveals 4 different clusters related to 4 different biological processes: direct phosphate metabolic processes, the response to oxidative stress, iron homeostasis and purine synthesis. If the full hit list is analyzed, the top 10 GO-terms of biological processes are all assigned to iron homeostasis related processes. Thus, the described algorithm may not only help to visualize the transcriptional networks and obtain statistical information on the networks, but also to group the genes to increase the likelihood of obtaining significant biological information from databases helping to identify multiple transcription factors and biological functions.

## DISCUSSION

Here we set out to define a way to cluster microarray data according to their expressional relationship and to obtain information on the significance of this clustering approach. The outlined approach allows obtaining good visualization of microarray results and provides solutions for several analysis problems. Initially, the clustering helps to shift the focus of the analysis towards the whole transcriptional response. The visual clustering versus the presentation in a table helps to understand the relationship of the hits towards each other, aids to visualize the potential transcriptional relationships, and further supports determining the transcription factors whose activation and inactivation results in the described responses. This might be hampered as long as all hits are used to search the relevant databases, but by using clusters this becomes more feasible.

Beyond that, the scoring function and the other parameters described enable a judgment of the quality of the network and allow evaluating the significance of the obtained results beyond the statistical significance of each individual hit. Despite their good performance in our study, parameters evaluating the network solely based on its included hits and connection numbers have shortcomings because they neglect all the genes, which are not part of the hit list. The CoRegScore instead connects the top100-hits used to construct the relevant network with co-regulated genes, which perform below the top100-list. Thus, it evaluates the significance of the analysis in the context of the large part of the experimental data set, which would be omitted if the network is just derived from the top100-hits.

Thus, analyses based on these three parameters - included hits, connections per hit and CoRegScore – may have internal evaluation criteria that possibly allow performing the full clustering analysis without the need to average biological replicates before. Instead, biological replicates could be individually analyzed by the described approach and then be compared based on the transcriptional clusters. These might be differentially affected in different biological replicates, which may be a realistic result analyzing transcriptional networks. To support this approach a server-based analysis tool was implemented, which is accessible from http://www.richterlab.de/Protocols/Protokolle-Software.htm. It performs an analysis on provided hit lists and returns the calculated network.

Our approach to clustering the top100-hits of any yeast microarray experiment would be the automated procedure performed by ClusterEx: 1.) The top100-hits can be determined from the array data by the user. 2.) These hits are assembled into a network matrix by ClusterEx using co-regulatory relationships from the SPELL database. 3.) Up to 50 connectors are determined from the interaction matrix and put into the network together with their connections. 4.) The determined connectors are evaluated to obtain the CoRegScore. If the CoRegScore is positive (significance is between 40 and 100), the clustering as visible in the network is significant. 5.) The network matrix is used in Cytoscape to visualize the relevant clusters together with the connectors, which improve the clustering due to their high connectivity. The "Edge-weighted spring embedded layout" according to Kamada and Kawai 38 is optimal for this visualization. 6.) Individual clusters are selected and GO-terms and transcription factors are found with PANTHER and YEASTRACT to guide further analyses and experiments.

Beyond the results presented for the yeast model organism it will also be interesting to see whether this approach can lead to similar results for higher organisms, such as *C. elegans*. Unlike yeast, nematodes consist of many different cells and cell types, each with its own transcriptional signature and, potentially, its specific response to developmental and environmental alterations.

## MATERIALS AND METHODS

### Datasets

The experimental datasets used in this study are based on Affymetrix Yeast Genome 2.0 microarray datasets consisting of 5815 expression values, which were evaluated previously 21. In short, yeast strains were transformed with a yeast expression plasmid harboring a polyglutamine-construct consisting of 56 glutamines. Likewise, yeast cells were transformed with a construct, leading to expression of an identically designed protein with either 30 or zero glutamine residues. Yeast cells were kept on transformation plates for three days and then harvested for RNA extraction and microarrays experiments. The microarrays experiments were performed and evaluated to yield the MAS5 and RMA-normalized values at the "Kompetenzzentrum für Fluoreszente Bioanalytik" at the Universität Regensburg.

As example datasets to test the described algorithm and parameters we used PCL-files from the sample set collection at http://spell.princeton.edu/spell/search/dataset_listing. All the sets we analyzed are also shown in the final figures.

### ClusterEx implementation

The full analysis procedure is performed automatically by the routine called ClusterEx, starting from loading the input files with the expression values to export of the network files and statistical parameters. MAS5- or RMA-processed datasets or PCL-files from SPELL can be used as input files. Alternatively, hit lists can be provided directly. Furthermore the number of wanted top-hits and the number of co-regulators can be set. Noise thresholds can be set and noise was considered throughout the study at MAS5-values below 15, a value about 30 % higher than the threshold, where statistical scattering of the results for the two conditions increased sharply 21. The C/C++ based routine was originally developed in the IDE Dev-C++ (Bloodshed Software) and further developed in the IDE Code::Blocks (The Code::Blocks Team). The routine is controlled from a graphical user interface developed in Visual Studio 2005 C# (Microsoft Corporation). Calculations were performed on a HP 250 notebook with 8 GB memory and Intel Celeron Dual-Core 2.16GHz processor. Analysis time for 100 hits with 20 co-regulators is in the range of 30 seconds. 

ClusterEx also can be run via a web interface accessible from http://www.richterlab.de/Protocols/Protokolle-Software.htm. The Apache2/PHP webserver connects from this page to the same routine running on a Raspbian Linux operating system. Analysis time for 30 hits with 10 co-regulators is currently in the range of 3 seconds and the web service will be upgraded periodically to allow calculations of more extensive networks.

### Network construction

Network construction was implemented in ClusterEx as described 21. In short, as shown in Figure 1 for each hit a number of renaked co-regulators were obtained from the SPELL webpage (http://spell.yeastgenome.org) , usually 20. The hit plus the 20 co-regulators yield 441 pairwise interactions (connections). These were collected in the interaction matrix for each hit, resulting in 44,100 connections for 100 hits if 20 co-regulators are used and 168,100 pairwise connections, if 40 co-regulators are used. These were sorted to ensure that each gene pair is only present once in the matrix, keeping the number of connections as a characteristic parameter for each gene pair. These numbers are later used to obtain the "connections per hit" as depicted in Figure 2B, S2B. The network together with the actual expression values for each of the genes included was exported as file for import and visualization in Cytoscape 23. All networks shown in this study were clustered using the ClusterEx-provided interaction matrix and the energy minimization function "Edge-weighted spring-embedded layout" from Cytoscape, which is based on an algorithm by Kamada and Kawai 38. After this only very limited graphical optimization was performed to better align overlapping nodes.

### Identification and evaluation of the best connector genes

Clusters as obtained from the top100-hits are unlikely to contain all the genes which belong to these clusters. Many of these instead are going to be part of the remaining 5,715 genes of the 5,815-sized full data set. These "missing genes" can be in parts obtained from the connection matrix, as they will be listed as co-regulators in the SPELL database and identifiable based on their large number of connections to the top100-hits. Identification of these best connected non-hit genes, called connectors, was then based on their performance within the co-regulation-matrix. A maximal number of connectors is given by the user and the threshold-number of connections required to be added was then determined by ClusterEx. If any gene had more connections to a hit gene then required by this threshold, it was maintained as connector. 

The connector genes increase the percentage of hits connected in the network and also the number of connections per hit considerably. They additionally enable the evaluation of a new parameter, describing the predictive quality of the network (CoRegScore). To obtain this CoRegScore for the connectors the experimental datasets were ranked with the highest up-regulated gene on top. The positions of the algorithmically derived connectors were then added up and used to calculate the score, which varies between 100 (perfect prediction) and -100 (entirely wrong prediction) with 0 being a useless prediction based on the equation:

**Figure:**
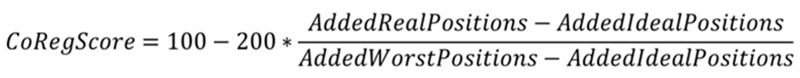


### Random networks

A procedure to obtain random hit lists was implemented for the genes included in the yeast FASTA genome library. 100 of these random sets were analyzed in the same manner as the experimental data sets. Genes with no entry in our SPELL-derived database were omitted from the analysis, leading currently to the exclusion of about 10-15% of the genes. The variation in results between the 100 different random hit lists was used to obtain standard deviations for the random hit lists. Selected random networks were visualized in Cytoscape to confirm that the connection parameters were calculated correctly. 

Furthermore, a procedure to generate random microarray expression datasets was implemented and here also 100 datasets were analyzed to evaluate the significance of the CoRegScore. The calculation was performed identically to the calculations on the experimental datasets and the results from the random sets were used to calculate the average CoReg Score for the random datasets and the standard deviation of this score. Due to the low connectivity of the random data sets often only few genes were included as connectors even though 50 were approached. This could lead to a higher standard deviation, but we refrained from excluding data sets, where only 1 or 2 connectors could be identified.

### Evaluation of isolated clusters by PANTHER and YEASTRACT

The networks generated by our approach were analyzed in Cytoscape. In order to scrutinize the biological relevance of the clustering approach, we analyze transcription factors regulating the isolated clusters and the biological processes the clustered genes are involved in. Genes which are grouped together and form isolated clusters are marked in the corresponding networks (Figure 1, 6, S3). Via the PANTHER web service we analyzed the biological processes the clusters are part of 35. Hereby the lists were analyzed for statistical overrepresentation of the gene ontology term "biological process". The hits were sorted according to their p-values on the website. The transcription factors regulating isolated clusters were analyzed via the YEASTRACT web service 36. We evaluated these lists for all transcription factors with physical binding evidence. For both analyses the three highest ranking hits were summarized in Table S1.

### Statistics 

Statistical analysis was performed for three parameters: the percentage of hits that could be connected in the network by at least one connecting gene. Furthermore, the average number of connections per hits was calculated. Based on this procedure the parameters used to construct the network, like the size of the initial top-hit list and the number of co-regulators obtained from the SPELL datasheets, were evaluated and refined. Also the CoRegScore was evaluated statistically based on the results from the random datasets.

The p-values for the differences in parameters between the networks derived from microarray data and random datasets were calculated based on the Z-score defined below.

**Figure:**
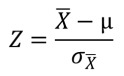


The values for µ and σ_X_ were obtained from the evaluation of 100 random datasets, with µ being the average value and σ_X_ being the standard deviation. The value for X originates from the evaluation of the experimental data set. The p-value, to show the probability of obtaining a higher reading compared to X from any random data set, can be obtained from the Z-score tables as "upper tailed tests" or "one-tail test" on http://www.socscistatistics.com/pvalues/normaldistribution.aspx.

### Programs and databases 

We used the SPELL (Serial Pattern of Expression Levels Locator) database for evaluating our datasets (http://spell.yeastgenome.org) . SPELL is a query-driven search engine for large gene expression microarray compendia. 

We used the PANTHER web service (http://amigo.geneontology.org/amigo/landing) for identifying GO-Terms within the isolated clusters. The PANTHER (Protein ANalysis THrough Evolutionary Relationships) Classification System was designed to classify proteins (and their genes) in order to facilitate high-throughput analysis 35. YEASTRACT (http://www.yeastract.com) was used to analyze transcription factors/regulators that potentially regulate an isolated cluster 36.

## SUPPLEMENTAL MATERIAL

Click here for supplemental data file.

All supplemental data for this article are also available online at http://microbialcell.com/researcharticles/construction-and-evaluation-of-yeast-expression-networks-by-database-guided-predictions/.
