# Supplementary material for: Construction and evaluation of yeast expression networks by database-guided predictions
Source: Microb Cell. 2016 Apr 21;3(6):236–47. doi: 10.15698/mic2016.06.505 (PMC5348991; doi:10.15698/mic2016.06.505)
Supplement: Supplementary file 1 [file mic-03-236-s01.pdf]

Supplemental Data

Construction and evaluation of yeast expression networks by database-guided predictions

Katharina Papsdorf[1,*], Siyuan Sima[1,*], Gerhard Richter[2], Klaus Richter[1, 3]

[1] Center of integrated protein science at the Technische Universität München, Department Chemie, Lichtenbergstr. 4, 85748 Garching

[2] address: gerhard.richter@richterlab.de.

[3] corresponding author: ,+49 89 289 13342, klaus.richter@richterlab.de,

* Contributed equally to this work

Table S1 Publicly available datasets used in this study.

Microarray Set	Collection	Column name Condition	Column in PCL-File
[25]	GSE5499_set0_family.pcl	HSF1 overexpression mutant vs. Empty vector , replicate 1	35
[24]	2010.Gasch00_HS25-37.flt.knn.avg.pcl	heat shock 40 min; src: time zero<->40 min	6
[34]	GSE32703_final.pcl	3 µM 1-NM-PP1 treatment for 40 min-_6	6
[35]	GSE8718_setA_family.pcl	5uM thiuram 1st	7
[36]	GSE9337_setA_family.pcl	87-86-5 Pentachlorophenol 1st	13
[37]	GSE9401_setA_family.pcl	7553-56-2 Iodine, 1 mM 2 h (time course) 1st H	22
[33]	GSE6302_set00_family.pcl	WT BY4741- glycerol vs. glucose at log phase- #1	1
[26]	GSE7525_set0_family.pcl	S.cerevisiae_alpha_factor_60min_repeat1	1
[38]	GSE11397_setA_family.pcl	wild-type +/- rapamycin, replicate 1	3
[27]	GSE23580_final.pcl	Wild type no vs high Pi conditions Replicate 1	49

Table S2 GO-Terms and transcription factors of isolated clusters compared to the full gene hit list.

Experiment	Cluster	GO-term	p-value		p-value	Transcription factor	p-value		p-value
Hsf1 overexpression	Hsf1 down 1	Hexose transport	1.16E-10	all genes	8.33E-01	Yap6	6.3679E-06	all genes	3.10893E-5
		Monosaccharide transport	1.16E-10		8.33E-01	Azf1	0.0010787		0.51814
		Carbohydrate transport	3.81E-09		8.28E-01	Sko1	0.001479		0.047044
	Hsf1 down 2	cellular respiration	1.05E-26	all genes	3.16E-04	Hap3	4.579E-08	all genes	0.023536
		generation of precursor metabolites and energy	4.46E-24		1.13E-02	Hap5	8.8838E-08		0.087763
		energy derivation by oxidation of organic compounds	6.76E-23		1.73E-03	Hot1	5.9425E-07		0.021446
	Hsf1 down 3	mitochondrial translation	4.38E-21	all genes	4.10E-01	Srd1	0.006218	all genes	n.r.
		mitochondrion organization	2.95E-16		1.00E00	Tos4	0.008018		0.021250
		translation	5.11E-14		1.00E00	War1	0.014052		n.r.
	Hsf1 down 4	cell wall organization or biogenesis	1.86E-01	all genes	1.00E+00	Mbp1	1.9632E-05	all genes	0.154289
		cell cycle	9.78E-01		1.00E+00	Skn7	1.0473E-4		0.045750
		n.r.	-		-	Yap6	0.000144		3.1089E-5
	Hsf1 up 1	Protein folding	1.03E-22	all genes	1.25E-03	Spt23	0	all genes	3.73503E-5
		Protein refolding	9.47E-16		2.45E-01	Hot1	1E-14		8.96635E-5
		Response to heat	4.19E-15		1.53E-02	Hsf1	7.4851E-11		0.0046591

Experiment	Cluster	GO-term	p-value		p-value	Transcription factor	p-value		p-value
Heat shock	Heat down-1	cytoplasmic translation	1.27E-15	all genes	1.00E00	Spt23	0	all genes	4.21814E-6
		translation	1.50E-11		1.00E+00	Ifh1	5.4592E-11		0.147782
		peptide biosynthetic process	1.82E-11		1.00E+00	Fhl1	4.9964E-08		0.895949
	Heat down-2	ribosome biogenesis	1.70E-76	all genes	1.29E-26	Sko1	0.010787	all genes	0.517678
		ribonucleoprotein complex biogenesis	6.93E-70		3.47E-23	Pog1	0.011101		0.040548
		rRNA processing	3.93E-54		2.21E-17	Spt23	0.011484		4.21814E-6
	Heat up-1	cellular carbohydrate metabolic process	2.75E-06	all genes	1.00E+00	Hot 1	0	all genes	4.2E-10
		carbohydrate metabolic process	6.07E-06		3.22E-01	Spt23	0		1.529120E-4
		trehalose metabolic process	9.67E-06		1.00E+00	Adr1	1E-15		1.007430E-4
Glucose vs Glycerole	Glucose down 1	cytoplasmic translation	3.97E-84	all genes	7.39E-24	Rap1	0	all genes	0.08508
		translation	8.47E-71		8.73E-20	Fhl1	0		5.64798E-7
		peptide biosynthetic process	2.21E-70		1.55E-19	Spt23	0		0
	Glucose down 2	organonitrogen compound metabolic process	1.54E-17	all genes	4.05E-27	Spt23	1.017E-05	all genes	0
		cellular amino acid metabolic process	8.39E-17		1.33E-06	Tos4	0.004078		0.31081
		organonitrogen compound biosynthetic process	1.01E-16		6.13E-27	Met28	0.007469		0.01428
	Glucose up 1	oxidation-reduction process	6.39E-21	all genes	9.93E-07	Adr1	0	all genes	6.780E-12
		cellular carbohydrate metabolic process	1.97E-13		3.22E-03	Hot1	0		1.00 E-13
		carbohydrate metabolic process	1.98E-11		7.52E-03	Sko1	0		2.125E-12

Experiment	Cluster	GO-term	p-value		p-value	Transcription factor	p-value		p-value
Phosphate starvation	Phosphate down 1	cellular amino acid biosynthetic process	7.33E-20	all genes	2.51E-01	Gcn4	5.0337E-05	all genes	0.999997
		organic acid biosynthetic process	3.08E-18		2.90E-01	Arg81	5.5629E-4		0.288809
		carboxylic acid biosynthetic process	3.08E-18		2.90E-01	Dal81	7.186E-4		0.012801
	Phosphate down 2	mitochondrial translation	2.74E-46	all genes	1.80E-10	Srd1	0.00392	all genes	n.r.
		mitochondrion organization	3.23E-42		1.03E-08	War1	0.01266		0.225037
		translation	3.13E-30		5.41E-04	Pog1	0.01559		0.251731
	Phosphate down 3	ATP metabolic process	1.53E-24	all genes	1.00E00	Hap3	0	all genes	0.035357
		purine ribonucleoside triphosphate metabolic process	3.29E-24		8.60E-01	Hap5	0		0.086808
		purine nucleoside triphosphate metabolic process	4.78E-24		1.00E00	Hap4	0		0.699949
	Phosphate up 1	phosphorus metabolic process	4.78E-05	all genes	3.29E-03	Pho4	4.5736E-08	all genes	8.1619E-4
		polyphosphate metabolic process	7.46E-05		3.39E-04	Met32	3.521 E-4		1.0711E-5
		dephosphorylation	4.70E-04		1.33E-01	Pdc2	0.002122		n.r.
	Phosphate up 2	response to oxidative stress	5.96E-10	all genes	1.00E00	Sko1	0	all genes	2.1695E-6
		cellular response to oxidative stress	5.68E-09		1.00E00	Hot1	0		3.79E-8
		trehalose metabolic process	3.41E-07		1.00E00	Spt23	1.082E-12		3.3663E-9
	Phosphate up 3	iron ion homeostasis	3.14E-23	all genes	4.10E-06	Put3	0	all genes	0.003282
		transition metal ion homeostasis	5.57E-23		1.71E-04	Aft1	9.055E-11		0.45898
		metal ion homeostasis	2.14E-21		1.13E-04	Aft2	6.3309E-09		0.09137

Experiment	Cluster	GO-term	p-value		p-value	Transcription factor	p-value		p-value
Phosphate starvation	Phosphate up 4	purine-containing compound biosynthetic process	1.08E-12	all genes	1.00E00	Cad1	2.026E-09	all genes	0.01664
		de novo' IMP biosynthetic process	1.97E-11		1.00E00	Yap5	5.7289E-09		0.12502
		purine nucleotide biosynthetic process	5.20E-11		1.00E00	Yap6	8.34E-05		1.94075E-5

Clusters were manually picked with Cytoscape [23]. The isolated clusters are indicated in the corresponding figure in the main manuscript. The genes within the clusters and the full hit list were analyzed. The GO-terms of the biological process were retrieved via the PANTHER algorithm [30] and the transcription factor via the YEASTRACT web service [31]. The p-values were retrieved within this analysis. 0= value below 0.0 E-15, n.r. = not retrieved.

Figure S1.

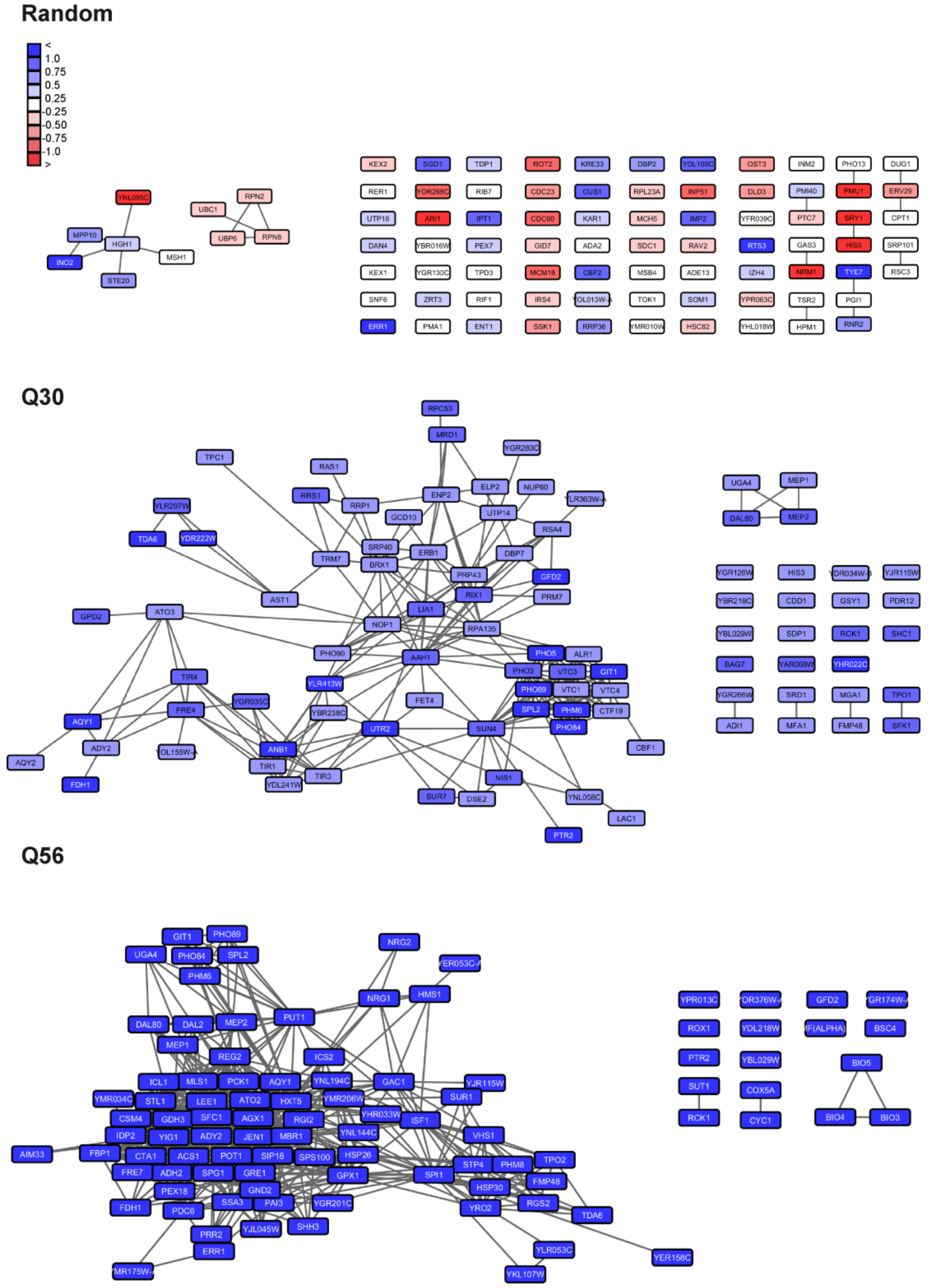

Figure S2

A

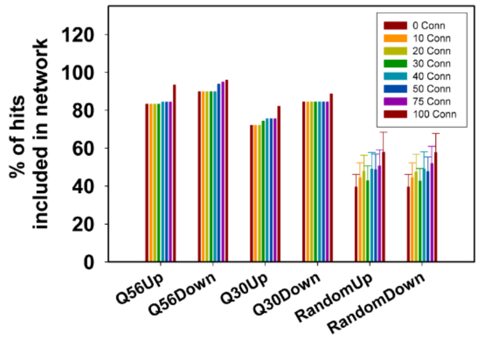
% of hits
included in network
0
20
40
60
80
100
120
0 Conn
10 Conn
20 Conn
30 Conn
40 Conn
50 Conn
75 Conn
100 Conn
Q56Up
Q56Down
Q30Up
Q30Down
RandomUp
RandomDown

B

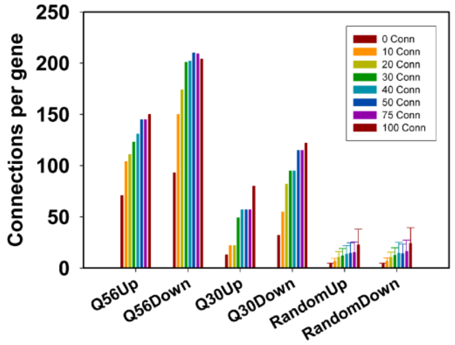
Connections per gene
0
50
100
150
200
250
0 Conn
10 Conn
20 Conn
30 Conn
40 Conn
50 Conn
75 Conn
100 Conn
Q56Up
Q56Down
Q30Up
Q30Down
RandomUp
RandomDown

Figure S3

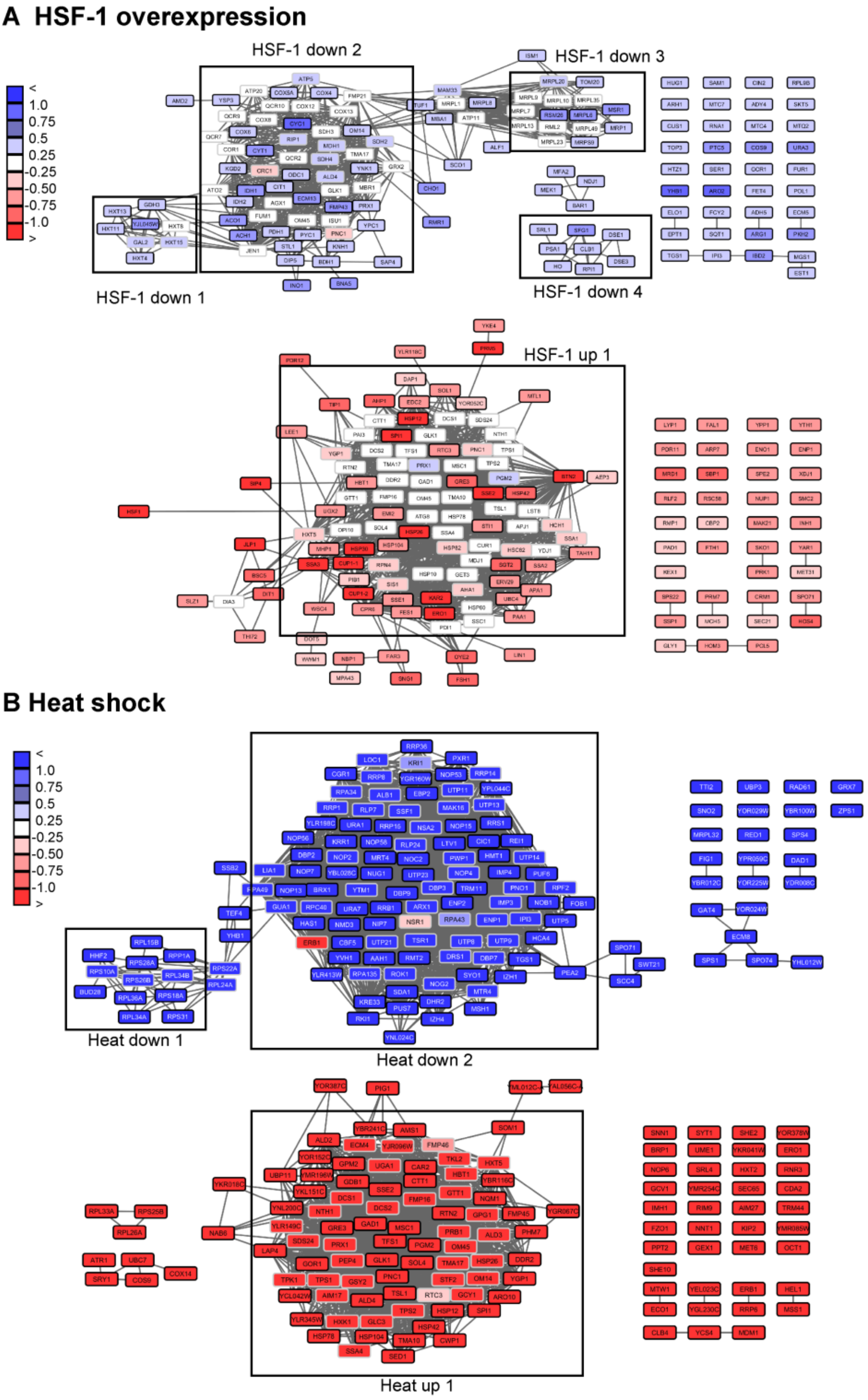
A HSF-1 overexpression
HSF-1 down 1
HSF-1 down 2
HSF-1 down 3
HSF-1 down 4
HSF-1 up 1
B Heat shock
Heat down 1
Heat down 2
Heat up 1
<
1.0
0.75
0.5
0.25
-0.25
-0.50
-0.75
-1.0
>

Figure S4

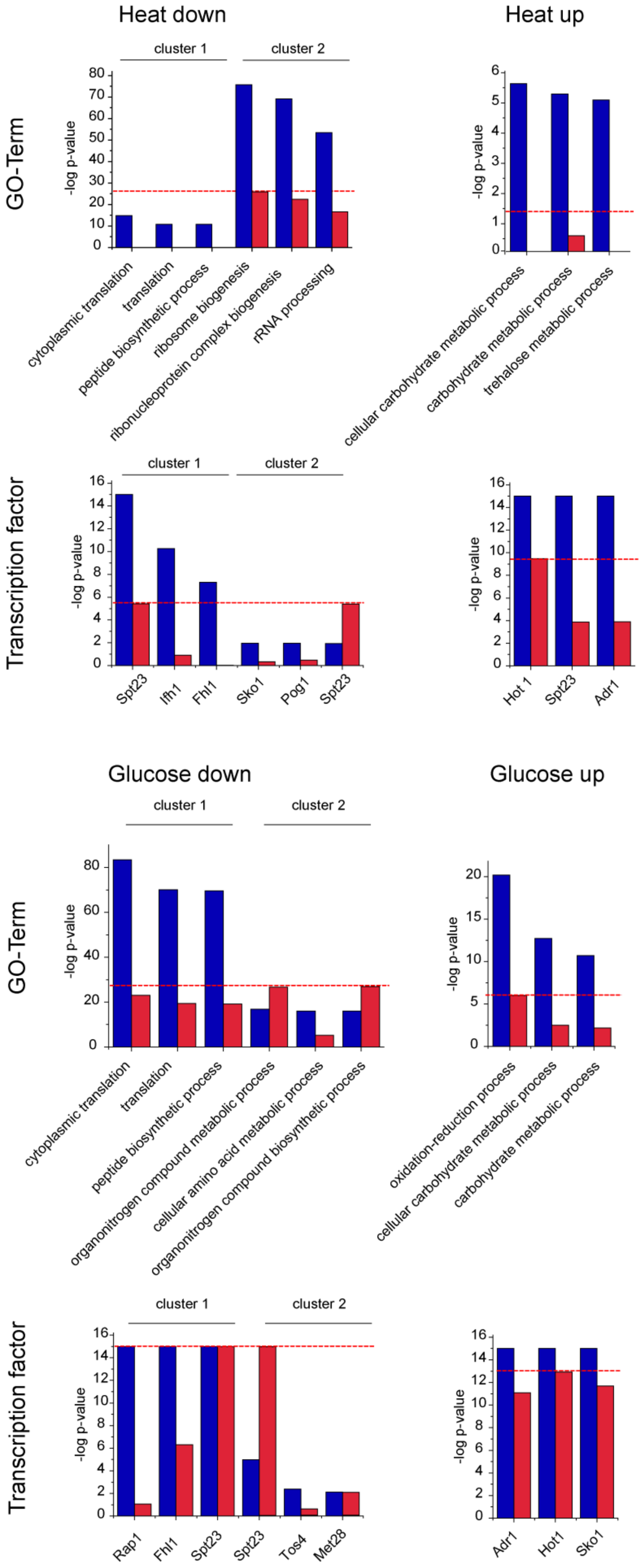
Heat down
Heat up
cluster 1
cluster 2
GO-Term
-log p-value
cytoplasmic translation
translation
peptide biosynthetic process
ribosome biogenesis
ribonucleoprotein complex biogenesis
rRNA processing
cellular carbohydrate metabolic process
carbohydrate metabolic process
trehalose metabolic process
Transcription factor
Spt23
Ifh1
Fhl1
Sko1
Pog1
Spt23
Hot 1
Spt23
Adr1
Glucose down
Glucose up
cluster 1
cluster 2
GO-Term
cytoplasmic translation
translation
peptide biosynthetic process
organonitrogen compound metabolic process
cellular amino acid metabolic process
organonitrogen compound biosynthetic process
oxidation-reduction process
cellular carbohydrate metabolic process
carbohydrate metabolic process
Transcription factor
Rap1
Fhl1
Spt23
Spt23
Tos4
Met28
Adr1
Hot1
Sko1

Figure S1. **Visualization of networks with different connectivity**.

The *top100* regulated genes of different datasets were processed in ClusterEx and visualized in Cytoscape. Connectivity is retrieved by obtaining 20 *coregulators* as described in Figure 1. The edge-weighted spring embedded layout is used to position highly connected genes in close proximity. Genes are colored is different shades of blue (downregulation) or red (upregulation) according to their experimental log differences. A) Random network of 100 genes. Random genes colored with expression values retrieved from the Q_{30} dataset were connected into a network. B) The *top100*-genes of the Q_{30} downregulated hit list were fit into an interconnected network. C) The *top100*-genes retrieved from the Q_{56} downregulated hit list were fit into an interconnected network and color coding was used as depicted in the log scalebar.

Figure S2. *Connectors* can be added to the network to evaluate clustering.

Strongly connected genes from the same transcriptional clusters which are not part of the initial hit list (called *connectors*) are included into the network. *top100*-hits were connected with 20 *coregulator*s and 10-100 *connectors* were obtained from the matrix and included into the network. A) Shown is the percentage of integration of the original hits into the network, depending on the number of *connectors* added to the network. B) Shown is the number of connections per gene including those between original hits and added *connectors*.

Figure S3. Visualisation of networks upon Hsf1-overexpression and heat stress in yeast.

The *top100* hits of different datasets were processed in ClusterEx and visualized in Cytoscape. Connectivity is retrieved by obtaining 20 *coregulator*s for each hit from SPELL and increased by including 50 *connectors* as described. The edge-weighted spring embedded layout is used to position highly connected genes in close proximity in Cytoscape. Genes are colored according to their log differences in the respective experiments. Clusters which are further analyzed for their GO-terms via PANTHER and for their transcription

factors via the YEASTRACT web service are marked with black boxes. A) Genes differentially regulated in *S. cerevisiae* upon Hsf-1 overexpression [25] were built into an interconnected network. The *top100*-hits (black frame) and 50 *connectors* (grey frame) were included into the network from the coregulation matrix. Upper panel: downregulation, lower panel: upregulation. B) Genes differentially regulated in *S. cerevisiae* upon heat stress [24] were built into an interconnected network. The *top100*-hits (black frame) and 50 *connectors* (grey frame) were derived from the matrix and included into their network. Upper panel: downregulation, lower panel: upregulation

Figure S4. Biological processes and transcription factors of isolated clusters.
Clusters are marked in Cytoscape as indicated in the corresponding figures (Figure 3, 8, 9). The gene-ontology term (GO-term) biological process of the genes (upper panel) and the transcription factors regulating the genes (lower panel) of the isolated clusters (blue) and the full gene hit list (red) are depicted. The full analysis is listed in Table S1 and described in the material and method section. The negative log of the p-value of the three highest ranking terms of the cluster analysis and the corresponding values of the full hit lists analysis are depicted. The red line represents p-value of the highest ranked GO-term or transcription factor analyzing the all genes in the network. - = analysis did not yield in any significant results.
